# Thomas Carwardine

**Published:** 1948

**Authors:** 


### Obituaries

### THOMAS CARWARDINE, M.S., F.R.C.S.

It was with great regret that we heard of the death of Thomas Carwardine on December 19th, 1947, at the age of eighty-two. He had had a long and distinguished association with the Bristol Royal Infirmary, where he was appointed House Surgeon and Senior Resident Officer on May 14th, 1895. Two years later, he became Assistant Surgeon on the death of Mr. Greig Smith, and Surgeon in January, 1906. He retired on reaching the age limit in 1926, and was Consulting Surgeon up to the time of his death.

His academic career was brilliant. After studying at University College, London, he entered the Middlesex Hospital. In the University of London he carried off the Gold Medal in Anatomy, in Surgery and in Diseases of Women.

He obtained the M.S. (London) and the F.R.C.S. (England) in 1894.

Carwardine was a fine operator, his anatomical knowledge was exact and his precision in handling his instruments was a delight to watch. He brought to the practice of Surgery the mind and hand of an artist. He drew excellently with pen or pencil and had a flair for decorative design. He designed several instruments, notably intestinal clamps, and in his student days a saccharometer for estimating sugar in the urine. He used to explain that as a clinical clerk, his chief admitted so many diabetics to his wards and required daily estimations of their glycosuria that he was compelled to invent a labour-saving instrument. The result is Carwardine's Saccharometer, still sold by the instrument makers.

He was President of the Bristol Medico-Chirurgical Society in 1925–26 and took as the subject of his Presidential address "Continuity and Change," in which he dealt with the then new discoveries of the instability of the atom and the continuity of life. He was not an active member of committees. At Faculty Meetings he was quiet, non-controversial but capable of talking sense at all times. In his younger days he had considerable taste for music and sang in Choral Societies. Photography was another of his interests.

His publications were not numerous and for the most part they appeared in this *Journal*. One on "Pericolitis" contained some admirable illustrations from his pen. In 1900 he published his only book, *Operative and Practical Surgery*.

In 1911 he married Professor Walker Hall's only child, May, who survives him and to whom we offer our deep sympathy.

